# Temporal trends in the prevalence of asthma and rhinoconjunctivitis in adolescents

**DOI:** 10.1590/S0034-8910.2015049005558

**Published:** 2016-01-12

**Authors:** Fernanda Agapito Pássaro Wilmer, Rosemeri Maurici, Carlos Alberto Kuntz Nazário, Kahio César Kuntz Nazário, Paula Fernanda Agapito Pássaro, Helena Elisa Piazza, Rennan Almir Bertoldi, Emílio Pizzichini, Márcia Margaret Menezes Pizzichini

**Affiliations:** I Programa de Pós-Graduação em Ciências Médicas. Universidade Federal de Santa Catarina. Florianópolis, SC, Brasil; II Curso de Medicina. Universidade Federal de Santa Catarina. Florianópolis, SC, Brasil; III Departamento de Clínica Médica. Faculdade de Medicina. Universidade do Sul de Santa Catarina. Palhoça, SC, Brasil

**Keywords:** Adolescent, Asthma, epidemiology, Rhinitis, epidemiology, Cross-Sectional Studies

## Abstract

**OBJECTIVE:**

To analyze the temporal trend of asthma and rhinoconjunctivitis prevalences as well as their symptoms in adolescents.

**METHODS:**

Two cross-sectional studies were conducted using the same methodology and questionnaire as was used for adolescents aged 12 to 14 years in the Brazilian city of Florianopolis, SC, Southern Brazil. Based on the international protocol of the International Study of Asthma and Allergies in Childhood (ISAAC) study, adolescents were evaluated in 2001 and 3,150 in 2012. The schools included in this study were the same as in the 2001 study. These schools were randomly selected after stratification by network (public and private) and geographic location. The total average percentage variation was estimated for the prevalence of asthma and rhinoconjunctivitis and their symptoms.

**RESULTS:**

The prevalence of reported asthma was 10.9% in 2001 and 14.8% in 2012, with an average variation of 2.8% in the period. The highest average variation in the period was observed among female adolescents (4.1%). In parallel a significant increase occurred in reported physician-diagnosed asthma, 7.3% in 2001 and 11,1% in 2012, with an annual variation of 4.5%. The largest increases in reported physician-diagnosed asthma were seen in female (5.9%) and male (4.5%) public school pupils. In addition, a significant increase in reported rhinoconjunctivitis occurred, with the average variation in the period being 5.2%. Reports of severe asthma symptoms remained unchanged during the period, while the annual variation for reported current wheezing (-1.3%) and wheezing during exercise (-1.2%) decreased.

**CONCLUSIONS:**

The results showed a significant increase in the annual average variation for asthma and rhinoconjunctivitis prevalence during the 2001 to 2012 period.

## INTRODUCTION

Asthma is the most common chronic respiratory disease for individuals during childhood and adolescence, the condition causes a high number of hospitalizations and has a high social cost.^17^ ^,^ ^22^ Asthma affects about 300 million individuals around the world, with the number of asthmatics expected to rise by 100 million by 2025.^9^ ^,^ ^14^ ^,^ ^19^ Considering the average prevalence of 10.0% of asthma sufferers that are diagnosed by a doctor, there are approximately 20 million asthmatics in Brazil.^19^ However, if we include the understanding that asthma symptoms are more prevalent than through medical diagnosis, this number may double, which indicates that the disease is currently being misdiagnosed and undertreated. These symptoms, besides having an effect on the quality of life for patients, increase costs that result from the disease, which are due to the higher number of emergency consultations and hospitalizations, as well as indirect costs from absenteeism.^2^ ^,^ ^11^ ^,^ ^18^

According to data from the Committee from the International Study of Asthma and Allergies in Childhood (ISAAC) study, Phase I, the prevalence of asthma among adolescents aged from 13 to 14 years ranged between 1.6% (Albania) and 28.8% (Australia), it is worth mentioning that the highest rates were found in western and industrialized countries.^4^ ^,^ ^5^ Brazil was the country with the fifth highest prevalence of asthma symptoms during a lifetime: 10.0%, 21.9% and 20.9%, in Sao Paulo, SP, Southeastern region, Porto Alegre, RS, Southern region, and Recife, PE, Northeastern region, respectively.^25^

In Florianopolis, SC, Southern region, Phase I of the 2001 ISAAC study^a^ involved approximately 4000 teenagers aged between 13 to 14 years. The prevalence of asthma symptoms experienced at some point during life was 11.9% in males and 10.0% in females. The prevalence of physician-diagnosed asthma was 8.5% in males and 6.2% in females.

Despite asthma and allergic rhinoconjunctivitis being commonly associated with each other, the prevalence of rhinoconjunctivitis in children has been studied to a lesser extent. Most of the information regarding rhinoconjunctivitis in other countries is related to hay fever and reports regarding its prevalence vary greatly among them.^3^ Allergic rhinoconjunctivitis affects approximately 400 million people around the world with the condition being more prevalent in developed countries.^4^ This disease can be understood as the most prevalent among chronic respiratory diseases (approximately 20.0% to 25.0% of the general population). Despite the disease having minor symptoms, it is among the 10 most frequent reasons for individuals seeking primary health care.^4^ During a recent study, which was performed in Brazilian 20 cities and used the ISAAC methodology, the average prevalence of allergic rhinoconjunctivitis among teenagers aged from 13 to 14 years was shown to range from 7.8% (rural region of Santa Maria, RS, Southern Brazil) and 21.1% (Salvador, BA, Northeastern Brazil).^8^

The prevalence of asthma is increasing in many parts of the world, which is especially true for developed countries.^5^ ^,^ ^23^ Despite the genetic factors being important, they are not likely to be able to explain these increases separately, which are attributed to the interaction between genetic and environmental factors. Environmental factors appear to be more relevant for determining the manifestations of this disease,^15^ ^,^ ^21^ whose etiology remains little understood, in spite of the considerable number of research projects that have been performed in the field.

Understanding the prevalence of asthma and its symptoms is relevant to guide health policies. Furthermore, measuring the temporal trends of asthma and its symptoms are important to gain a better understanding of its epidemiology.

The aim of this study was to analyze the temporal trend of asthma and rhinoconjunctivitis prevelance in addition to the condition’s symptoms in adolescents.

## METHODS

This study is of a descriptive, analytical epidemiological nature, which compares two cross-sectional studies that investigated the prevalence of asthma, its symptoms, and rhinoconjunctivitis in 12 to 14 year old adolescents, who were enrolled in public and private schools in Florianopolis, in 2001 (n = 4,114) and 2012 (n = 3,150).

The sample is founded on the international protocol from the ISAAC study,^10^ which states that the sample should include between 1,000 and 3,000 adolescents from this age group, with a estimated non-response rate of 20.0%. These figures were calculated to make analysis of the subgroups possible and enable the drawing of comparisons with other national and international centers in which the same methodology was applied. In addition, the figures showed a difference 5.0% in the prevalence of asthma and its symptoms, with a 95.0% detection power and 1% significance level. As regards the severity of the symptoms, the detection power for differences between 3.0% and 5.0% is 90.0%, with a 1% significance level.

The same randomly selected schools from the 2001 study were included in this study. Some of the schools from the previous study had closed and were therefore replaced by other randomly chosen schools, while maintaining the same network and education sector. These schools were chosen using a table of random numbers following a stratification that was based on network (private and public) and geographic location (seven educational sectors), while keeping the proportional nature of the number of students enrolled in each region intact. The data were collected by suitably qualified medical students and a psychologist, between May and July 2012.

The same ISAAC phase III questionnaire was used in this study, which is identical to that used in phase I, with added questions that refer to asthma risk factors. The questionnaire is self-administered, validated and structured so as to measure differences in the prevalence of asthma, rhinoconjunctivitis and eczema in countries with differing languages and cultures. In addition to demographic information, the questionnaire included questions regarding the previous (at some point during the individual’s life) and current (within the last 12 months) presence of asthma and allergic rhinoconjunctivitis, in addition to the symptoms of both diseases. This version of the questionnaire was adapted to contain additional information about the medical diagnosis of asthma, tobacco use and contact with domestic animals. This same procedure has been adopted in several publications in which the ISAAC questionnaire was used. Adjustments were permitted, provided that the central part of the questionnaire remains unchanged.

The prevalence of asthma symptoms was based on answers to the following variables: (a) wheezing in the previous 12 months (current wheezing); (b) frequent wheezing episodes in the previous 12 months; (c) dry nighttime coughing in the previous 12 months; and (d) wheezing during exercise in the previous 12 months. Severe symptoms of asthma were defined as a positive response to current wheezing, four or more episodes of wheezing, waking up at night one or more times per week due to wheezing, or wheezing having an effect on speech. The prevalence of asthma was reported based on the answer to the following question: “Have you ever had asthma?”.

The prevalence of rhinoconjunctivitis was estimated based on positive responses to the following two questions: “In the last 12 months, when not suffering from cold or flu, have you had any problems such as sneezing or a runny or blocked nose?” and “In the previous 12 months, was this nasal problem accompanied by itchy and watering eyes?”.

The results of the study were grouped into frequencies or proportions; the significance of the differences between the groups and subgroups was established using the Chi-square test where indicated.

The total average annual percentage variation was estimated with the eleventh root, where p2001 p2012 are the prevalences calculated for 2001 and 2012, respectively.^24^


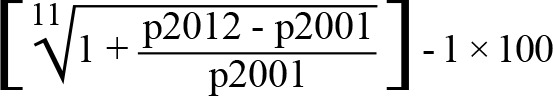


The Wald test for linear trend was used to verify the temporal trend. A 5% significance level was adopted (p < 0.05) and all tests were two-tailed.

This study was approved by the Human Research Ethics Committee at the Universidade Federal de Santa Catarina (Process 2397/476735 /2008). All participants and their legal guardians signed an free and informed consent form.

## RESULTS

The study population was made up of adolescents who were selected in 2001 (n = 4,114) and in 2012 (n = 3,150). The response rate was 75.5% in 2001 and 81.3% in 2012. With the exception of a higher percentage of 12-year-old adolescents in 2012, the other demographic characteristics of each year were similar. However, the analysis of the study’s outcomes of interest did not differ among the ages of 12, 13 and 14 years between 2001 and 2012.

The average annual prevalence of individuals who reported having had asthma at some point and medical diagnosis of asthma (Table 1) varied. Nevertheless, the increase in the annual variation of asthma diagnosis was higher than the number of individuals who reported having asthma at some point (+4.5% *versus* +2.8%, respectively). In contrast, there was a significant reduction in annual variation regarding the prevalence of wheezing in the previous year and wheezing during exercise. The prevalence of severe asthma symptoms remained unchanged during this period.

**Table 1:** Prevalence and annual average variation of asthma at some point during life, medical diagnosis, severe symptoms during the previous year and having reported rhinoconjunctivitis. Florianopolis, SC, Southern Brazil, 2001 to 2012.

Variable	2001 (n = 3,053)	2012 (n = 2,563)	Annual variation (%)	p*
Asthma at some time	10.9	14.8	+ 2.8	< 0.001
Medical diagnosis of asthma	7.3	11.1	+4.5	< 0.001
Current wheezing	18.8	16.2	-1.3	0.030
Severe symptoms of asthma	6.7	6.7	0	1.000
Wheezing during exercise	21.4	18.7	-1.2	0.010
Dry nighttime coughing	35.6	43.7	+1.9	< 0.001
Rhinoconjunctivitis	20.4	35.4	+5.2	< 0.001

* Chi-square test.

Much of the variability in the prevalence of reported asthma and medical diagnosis was due to the significant increase in the average annual variation in the female gender during the period. In addition, the prevalence of dry nighttime coughing and reported rhinoconjunctivitis was significantly higher in females over the two study periods (Table 2).

**Table 2:** Prevalence and annual average variation of asthma at some point during life, medical diagnosis, symptoms during the previous year and reported rhinoconjunctivitis in adolescents aged from 12 to 14 years, according to gender. Florianopolis, SC, Southern Brazil, 2001 to 2012.

Variable	200	2012	Annual variation (%)	p*
Asthma at some time				
Male	11.9	14.1	+1.6	0.090
Female	10.0	15.6	+4.1	< 0.001
Medical diagnosis of asthma				
Male	8.5	10.6	+2.0	0.070
Female	6.2	11.7	+5.9	< 0.001
Current wheezing				
Male	17.8	14.8	-1.7	0.030
Female	19.6	17.5	-1.0	0.200
Wheezing during exercise				
Male	18.8	18.2	-0.3	0.600
Female	23.7	19.1	-1.9	0.003
Dry nighttime coughing				
Male	30.1	36.2	+1.7	0.001
Female	40.6	51.0	+2.1	< 0.001
Rhinoconjunctivitis				
Male	14.9	26.6	+5.4	< 0.001
Female	20.4	35.4	+5.1	< 0.001

* Chi-square test.

The prevalence of current wheezing, severe asthma symptoms, wheezing during exercise, dry nighttime coughing and reported rhinoconjunctivitis was significantly higher in adolescents who had had it at some point in their lives. Most of the participants who reported having experienced asthma had this diagnosis confirmed by a physician, both in 2001 and in 2012 (Table 3).

**Table 3:** Prevalence of asthma symptoms, reported rhinoconjunctivitis and medical diagnosis of asthma in adolescents aged from 12 to 14 years old, who reported having had asthma. Florianopolis, SC, Southern Brazil, 2001 to 2012.

Asthma at some time	2001			2012		

	Yes (%)	No (%)	p*	Yes (%)	No (%)	p*
n	333	2,720		380	2,183	
Wheezing in the previous 12 months	50.8	14.9	< 0.001	45.8	11.0	< 0.001
Severe symptoms of asthma	25.4	4.4	< 0.001	24.2	3.7	< 0.001
Wheezing during exercise	48.0	18.1	< 0.001	44.2	14.2	< 0.001
Dry nighttime coughing	52.6	33.5	< 0.001	59.1	41.0	< 0.001
Rhinoconjunctivitis	32.7	15.9	< 0.001	45.8	28.5	< 0.001
Medical diagnosis of asthma	60.7	0.8	< 0.001	68.4	1.1	< 0.001

* Chi-square test.

**Table 4:** Prevalence and average annual variation in the reporting of having asthma at some point in life, medical diagnosis and symptoms of asthma over the previous year and reported rhinoconjunctivitis according to the educational network (public and private). Florianopolis, SC, 2001 to 2012.

Variable	2001 (%)	2012 (%)	Annual variation (%)	p*
Asthma at some time				
Public	9.7	13.5	+3.1	< 0.001
Private	13.7	17.6	+2.3	0.030
Medical diagnosis of asthma				
Public	6.1	9.9	+4.5	< 0.001
Private	10.1	13.5	+2.8	0.030
Current wheezing				
Public	18.5	14.0	-2.5	< 0.001
Private	19.5	20.5	+0.5	0.600
Wheezing during exercise				
Public	21.8	18.5	-1.7	0.009
Private	20.4	19.2	-0.5	0.500
Dry nighttime coughing				
Public	35.2	40.9	+1.4	< 0.001
Private	36.4	49.5	+2.8	< 0.001
Rhinoconjunctivitis				
Public	16.5	27.2	+4.6	< 0.001
Private	20.6	38.9	+6.9	< 0.001

* Chi-square test.

Despite the significant differences between the public and private networks in both periods, the average annual variation in the various prevalences, with the exception of rhinoconjunctivitis, was similar between the two networks.

## DISCUSSION

The results showed a significant increase in the average annual variation in the prevalence of asthma and rhinoconjunctivitis in adolescents aged 12 to 14 years old in the period. This result was mainly due to the increased number of female participants. The number of asthma cases being medically diagnosed in girls and in both educational networks (private and public) increased. In contrast, the prevalence of severe asthma symptoms remained unchanged. The prevalence of current wheezing, wheezing during exercise, dry nighttime coughing, severe symptoms of asthma, rhinoconjunctivitis and medical diagnosis of asthma was significantly higher in adolescents who reported having asthma at some point in their lives than those who did not. These final results confirm the consistency and reliability of the responses related to asthma and its symptoms over the two study periods.

Solé et al^20^ compared the prevalence of asthma and rhinoconjunctivitis in phases I and III of the ISAAC study and analyzed the changes of these conditions in adolescents between 13 and 14 years of age in five Brazilian cities (Recife, Salvador, Sao Paulo, Curitiba and Porto Alegre). These authors found a decreasing tendency in the prevalence of current wheezing, but not in the prevalence of asthma or rhinoconjunctivitis. The reduced prevalence of reported asthma at some point during life and speech-impeding wheezing (one of the severe symptoms of asthma) was more consistent in adolescents from Porto Alegre. Current wheezing in adolescents from all the studied sites decreased, the exceptions being Curitiba and Recife.

Phases I and III of the ISAAC study^4^ showed large variations in prevalence, not only among countries, but also within the same country, in which many centers performed the study. In addition, a more detailed study on the global prevalence of asthma symptoms at Phases I and III of the ISAAC study highlighted that the variability in prevalence, found among the various countries, was due to the larger annual increase in the prevalence of asthma and its symptoms in countries where this prevalence was lower during Phase I; and a stabilization or even reduction in countries where this prevalence was higher.^16^

One possible explanation for the differences in the prevalence of asthma and its symptoms in Florianopolis and the other Brazilian cities studied by Solé et al,^20^ from a study example made by Pearce et al,^16^ is that, in those capitals, these prevalences were already higher than those reported in Florianopolis. Other possible explanations, regarding the considerable increase in the prevalence of asthma and rhinoconjunctivitis observed in our study, are represented by the interval between Phases I and III of the ISAAC study, as well as by potential interference from local factors. Further investigation is deserved regarding the factors that may relate to socioeconomic changes, greater attention to the symptoms of both diseases by patients, families and doctors, or by other factors that were not detected during this study. The 10-year interstice between the two studies can be characterized by the increased supply and access to health services, in addition to the substantial increase in population density, whose repercussions are most notable in the environmental sphere.

Wehrmeister et al,^24^ while employing prevalence data from the national household sample survey, from 1998 to 2008, studied the trend of asthma prevalence in Brazilian children and adolescents aged 10 to 19 years. The prevalence of asthma in this group of adolescents increased 2.2%. The greatest increments were observed among boys and among rural residents. The results of this study are different from those mentioned above, since the largest annual increase in the prevalence of asthma during the period was with girls. The different methodological approaches between the two studies might explain these discrepancies.

From another Brazilian study that used the ISAAC methodology in adolescents aged 13 and 14 years, a higher prevalence of asthma symptoms in females was reported.^12^ Several factors are related to the association of asthma in adolescent and female individuals, including genetic factors, maternal-fetal interactions, maturation of the immune system, hormonal changes and psychosocial and environmental influences that are specific to each gender.^1^ ^,^ ^12^

Despite the significant increase in reported asthma at some point in an individual’s life and in the medical diagnosis of asthma observed during this study, we observed decrease in the prevalence of current wheezing (in the previous 12 months) and wheezing during exercise in the global analysis of the results, as when comparing the distribution of this symptom by gender and education network. The significant decrease of these symptoms, without taking the reduction of asthma diagnosis into account, can be related to asthma medicines being more available from the public health network, as well as access to health services being greater over the previous decade. Similarly, the stabilizing prevalence of severe asthma symptoms could be a result of the same factors.

The global trend for symptoms and for the presence of rhinoconjunctivitis, using data from Phase I and III of the ISAAC study, was investigated by Asher et al.^4^ In Brazil, these authors found that the average prevalence was 16.2% during Phase I and 15.8% during Phase III of the study. More specifically, there were important variances in several of the evaluated cities, with the prevalence of rhinoconjunctivitis being 25.0% and 21.1% in Salvador, 11.3% and 14.2% in Recife, 12.6% and 15.6% in Sao Paulo, 14.1% and 17.0% in Curitiba and 17.6% and 14.2% in Porto Alegre during phases I and III, respectively.^8^ The results of this study showed an increase in the annual variation that was superior to that reported in the literature.^6^ ^,^ ^7^ These differences may be due to the time gap, on years, between the periods studied or the socio-economic changes.

Asthma and rhinoconjunctivitis are conditions that are commonly associated with each other. The main etiological factors for both are atopy, exposure to allergens inside and outside the home or at work, in addition to factors such as: geographical variations, genetics, lifestyle, industrialization, poverty, living with animals, passive smoking, humidity and viral infection during the early years of life.^13^ 32.7% and 45.8% of the adolescents from this study, who reported having had asthma at some point in 2001 and 2012, respectively, also reported having rhinoconjunctivitis.

Several aspects of this study make its results strong: use of a standardized methodology for the two research periods; high response rate; strict verification of the data obtained as well as for the methodology used to acquire it.

One possible limitation of this study was that 12-year-old individuals were included, since almost all of the ISAAC studies report results for teenagers aged between 13 and 14 years old. The decision to include 12-year-old students in the study, who were due to turn 13 during the study year, was maintained for both periods.

However, this seems to be less important than the sample’s representativeness, since the respondents themselves are not the same. In other reports from Phases I and III of the ISAAC study, at some centers, the schools interviewed for Phase III were completely different from those for Phase I.^4^

The results of this study show that, during the period, a significant annual increase in the prevalence of reported asthma and rhinoconjunctivitis and their symptoms in adolescents between 12 and 14 years of age occurred in Florianopolis from 2001 to 2012. Medical diagnoses of asthma increased; however these were not accompanied by an increase in symptoms that are considered severe, which may explain the improvement in the quality of medical care regarding the diagnosis and therapeutic aspects. However, this evolution is above what is observed in other countries and in Brazil itself, which suggests that local factors may play a part, the way in which this occurs deserves investigation during future studies.

## Footnotes

a Piazza HE. Prevalência de sintomas de asma e rinite em adolescentes escolares da cidade de Florianópolis [dissertation]. Florianópolis (SC): Universidade Federal de Santa Catarina; 2001.
