# Supplementary material for: Temporal trends in the prevalence of asthma and rhinoconjunctivitis in adolescents
Source: Rev Saude Publica. 2016 Jan 12;49:94. doi: 10.1590/S0034-8910.2015049005558 (PMC4716651; doi:10.1590/S0034-8910.2015049005558)
Supplement: Translation

# Translation: Evolução temporal na prevalência de asma e rinoconjuntivite em adolescentes

**Authors:** Fernanda Agapito Pássaro Wilmer, Rosemeri Maurici, Carlos Alberto Kuntz Nazário, Kahio César Kuntz Nazário, Paula Fernanda Agapito Pássaro, Helena Elisa Piazza, Rennan Almir Bertoldi, Emílio Pizzichini, Márcia Margaret Menezes Pizzichini

**Affiliations:** I Programa de Pós-Graduação em Ciências Médicas. Universidade Federal de Santa Catarina. Florianópolis, SC, Brasil; II Curso de Medicina. Universidade Federal de Santa Catarina. Florianópolis, SC, Brasil; III Departamento de Clínica Médica. Faculdade de Medicina. Universidade do Sul de Santa Catarina. Palhoça, SC, Brasil

**Keywords:** Adolescente, Asma, epidemiologia, Rinite, epidemiologia, Estudos Transversais

## Abstract

**OBJETIVO:**

Analisar a tendência temporal da prevalência de asma e de rinoconjuntivite e seus sintomas em adolescentes.

**MÉTODOS:**

Foram realizados dois estudos transversais utilizando a mesma metodologia e o mesmo questionário em adolescentes de 12 a 14 anos em Florianópolis, SC. Fundamentado no protocolo internacional do estudo *International Study of Asthma and Allergies in Childhood *(ISAAC), foram avaliados 4.114 adolescentes em 2001 e 3.150 em 2012. As escolas estudadas foram as mesmas de estudo anterior de 2001, selecionadas aleatoriamente após estratificação por rede (pública e privada) e por localização geográfica. A variação percentual anual média total foi estimada para a prevalência de asma e seus sintomas e para rinoconjuntivite.

**RESULTADOS:**

A prevalência de relato de asma foi de 10,9% em 2001 e de 14,8% em 2012, com variação média no período de 2,8%. A maior variação média no período ocorreu entre os adolescentes do sexo feminino (4,1%). Houve aumento significativo no relato de diagnóstico médico de asma, de 7,3% em 2001 para 11,1% em 2012, com variação anual de 4,5%. Os maiores aumentos no relato de diagnóstico médico de asma ocorreram em alunos do sexo feminino (5,9%) e masculino (4,5%) da rede pública. Houve incremento expressivo no relato de rinoconjuntivite, com variação média no período de 5,2%. O relato de sintomas de asma grave permaneceu inalterado e houve decréscimo na variação anual no período no relato de sibilos atuais (-1,3%) e de sibilos aos exercícios (-1,2%).

**CONCLUSÕES:**

Os resultados mostraram incremento significativo na variação anual média da prevalência de asma e de rinoconjuntivite no período de 2001 a 2012.

## INTRODUÇÃO

A asma é a doença respiratória crônica mais comum da infância e da adolescência, com elevado número de internações e alto custo social.^17^ ^,^ ^22^ A asma acomete cerca de 300 milhões de indivíduos em todo o mundo, com acréscimo previsto de 100 milhões de asmáticos até 2025.^9^ ^,^ ^14^ ^,^ ^19^ Considerando prevalência média de 10,0% de asma diagnosticada por médico, existem aproximadamente 20 milhões de asmáticos no Brasil.^19^ Contudo, se levarmos em conta que os sintomas de asma são mais prevalentes do que o diagnóstico médico, esse número pode dobrar, o que indica subdiagnóstico e, consequentemente, subtratamento da doença. Esses sintomas, além de afetarem a qualidade de vida dos pacientes, aumentam os custos com a doença, decorrentes de maior número de consultas de emergência e de hospitalizações, e de custos indiretos devido ao absenteísmo.^2^ ^,^ ^11^ ^,^ ^18^

Segundo dados do Comitê do estudo *International Study of Asthma and Allergies in Childhood *(ISAAC) Fase I, a prevalência de asma entre adolescentes de 13 a 14 anos variou entre 1,6% (Albânia) e 28,8% (Austrália), cujas taxas mais elevadas foram encontradas nos países ocidentais e industrializados.^4^ ^,^ ^5^ O Brasil foi o quinto país com maior prevalência de sintomas alguma vez na vida: 10,0%, 21,9% e 20,9%, em São Paulo, SP, Porto Alegre, RS, e Recife, PE, respectivamente.^25^

Em Florianópolis, SC, a Fase I do estudo ISAAC, realizada em 2001,^a^ envolveu aproximadamente quatro mil adolescentes de 13 a 14 anos. A prevalência de asma alguma vez na vida foi de 11,9% no sexo masculino e de 10,0% no feminino. A prevalência de asma diagnosticada por médico foi de 8,5% e 6,2% nos sexos masculinos e femininos, respectivamente.

Embora asma e rinoconjuntivite alérgica estejam comumente associadas, a prevalência de rinoconjuntivite em crianças é menos estudada. A maioria das informações sobre rinoconjuntivite, em outros países, está relacionada à febre do feno e os relatos das taxas de prevalência variam muito entre eles.^3^ A rinoconjuntivite alérgica acomete aproximadamente 400 milhões de pessoas no mundo e sua prevalência é maior nos países desenvolvidos.^4^ Pode ser considerada a doença de maior prevalência entre as doenças respiratórias crônicas (cerca de 20,0% a 25,0% da população em geral). Embora com sintomas de menor gravidade, está entre as 10 razões mais frequentes de atendimento em atenção primária em saúde.^4^ Estudo recente realizado em 20 cidades brasileiras, usando a metodologia ISAAC, mostrou que a prevalência média de rinoconjuntivite alérgica entre adolescentes de 13 a 14 anos variou entre 7,8% (região rural de Santa Maria, RS) e 21,1% (Salvador, BA).^8^

A prevalência da asma vem aumentando em várias partes do mundo, principalmente nos países desenvolvidos.^5^ ^,^ ^23^ Os fatores genéticos, embora importantes, provavelmente não explicam isoladamente esses aumentos, atribuídos à interação entre fatores genéticos e ambientais. Os fatores ambientais parecem ter maior relevância na determinação das manifestações dessa doença,^15^ ^,^ ^21^ cuja etiologia permanece pouco compreendida, a despeito de considerável número de pesquisas na área.

Conhecer a prevalência da asma e seus sintomas é relevante para orientar políticas de saúde. Além disso, medidas de tendências temporais na prevalência da asma e seus sintomas são importantes para a melhor compreensão da sua epidemiologia.

O presente estudo teve por objetivo analisar a tendência temporal da prevalência da asma e da rinoconjuntivite e seus sintomas em adolescentes.

## MÉTODOS

Estudo epidemiológico, descritivo e analítico, que compara dois estudos transversais que investigaram a prevalência de asma, seus sintomas, e a rinoconjuntivite, em adolescentes de 12 a 14 anos, matriculados nas escolas da rede pública e privada de Florianópolis, em 2001 (n = 4.114) e 2012 (n = 3.150).

Fundamentado no protocolo internacional do estudo ISAAC,^10^ a amostra deveria incluir entre 1.000 e 3.000 adolescentes dessa faixa etária, prevendo-se taxa de não resposta de 20,0%. Esses números foram calculados para permitir a análise de subgrupos e estabelecer comparações com outros centros nacionais e internacionais em que a mesma metodologia foi aplicada. Além disso, permitem distinguir diferença de 5,0% na prevalência de asma e seus sintomas com poder de detecção de 95,0% e nível de significância de 1%. Quanto à gravidade dos sintomas, o poder de detecção para diferenças entre 3,0% a 5,0% é de 90,0%, com nível de significância de 1%.

As escolas incluídas foram as mesmas do estudo de 2001, selecionadas aleatoriamente. Algumas escolas do estudo anterior foram fechadas e, portanto, foram substituídas por outras, escolhidas aleatoriamente, preservando-se a mesma rede e setor de ensino. Essa escolha foi feita utilizando-se uma tabela de números randômicos após estratificação por rede (pública e privada) e por localização geográfica (sete setores de ensino), guardando a proporcionalidade do número de alunos matriculados em cada região. Os dados foram coletados por alunos de medicina e por uma psicóloga, devidamente capacitados, de maio a julho de 2012.

Utilizou-se o mesmo questionário ISAAC Fase III, idêntico ao utilizado na Fase I, acrescido de perguntas relevantes aos fatores de risco para asma. O questionário é estruturado, autoaplicável e validado para aferir diferenças de prevalência de asma, rinoconjuntivite e eczema em países com língua e culturas diferentes. Além de informações demográficas, o questionário incluiu perguntas sobre a presença anterior (alguma vez) e atual (nos últimos 12 meses) de asma e rinoconjuntivite alérgica, além dos sintomas de ambas as doenças. A versão do questionário utilizado foi adaptada para conter informações adicionais sobre o diagnóstico médico de asma, tabagismo e contato com animais domésticos. Esse procedimento tem sido adotado em diversas publicações em que o questionário ISAAC foi utilizado. Adaptações são permitidas, desde que a parte central do questionário permaneça inalterada.

A prevalência de sintomas de asma foi baseada nas respostas às seguintes variáveis: (a) sibilos nos últimos 12 meses (sibilos atuais); (b) episódios frequentes de sibilância nos últimos 12 meses; (c) tosse seca noturna nos últimos 12 meses; e (d) sibilos aos exercícios nos últimos 12 meses. Sintomas graves de asma foram definidos como resposta positiva a sibilos atuais, a quatro ou mais episódios de sibilos, despertar noturno uma ou mais vezes por semana por causa dos sibilos, ou sibilos afetando a fala. A prevalência de asma relatada foi baseada na resposta à pergunta: “Alguma vez você teve asma?”.

A prevalência de rinoconjuntivite foi estimada pelas respostas positivas a duas perguntas: “Nos últimos 12 meses, você teve problemas como espirro ou nariz escorrendo ou trancado, quando não está com gripe ou resfriado?” e “Nos últimos 12 meses, este problema no seu nariz foi acompanhado por coceira e lacrimejamento nos olhos?”.

Os resultados do estudo foram agrupados por meio de frequências ou proporções; a significância das diferenças entre grupos e subgrupos foi estabelecida pelo uso do teste do Qui-quadrado onde indicado.

A variação percentual anual média total foi estimada com a raiz undécima em que p2001 e p2012 são as prevalências calculadas para 2001 e 2012, respectivamente.^24^



O teste de Wald para tendência linear foi utilizado para verificar a tendência temporal. O nível de significância considerado foi de 5% (p < 0,05) e todos os testes foram bicaudados.

O estudo foi aprovado pelo Comitê de Ética em Pesquisa com Seres Humanos da Universidade Federal de Santa Catarina (Processo 2397/476735/2008). Os participantes e seus responsáveis assinaram o termo de consentimento livre e esclarecido.

## RESULTADOS

A população do estudo foi composta pelos adolescentes selecionados em 2001 (n = 4.114) e pelos selecionados em 2012 (n = 3.150). A taxa de resposta em 2001 foi de 75,5% e em 2012 foi de 81,3%. Com exceção de percentual maior de adolescentes de 12 anos na amostra de 2012, as demais características demográficas de cada ano foram similares. Contudo, a análise dos desfechos de interesse do estudo não diferiu entre as idades de 12, 13 e 14 anos entre 2001 e 2012.

Houve variação anual média na prevalência de relato de asma alguma vez e de diagnóstico médico de asma (Tabela 1). No entanto, o aumento na variação anual de diagnóstico de asma foi superior ao de relato de asma alguma vez (+4,5% *versus* +2,8%, respectivamente). Em contraste, houve redução na variação anual significativa nas prevalências de sibilos no último ano e sibilos aos exercícios. A prevalência de sintomas graves de asma permaneceu inalterada durante o período.

**Tabela 1:** Prevalência e variação anual média de asma alguma vez, diagnóstico médico, sintomas graves no último ano e relato de rinoconjuntivite. Florianópolis, SC, 2001 a 2012.

Variável (%)	2001 (n = 3.053)	2012 (n = 2.563)	Variação anual (%)	p*
Asma alguma vez	10,9	14,8	+2,8	< 0,001
Diagnóstico médico de asma	7,3	11,1	+4,5	< 0,001
Sibilos atuais	18,8	16,2	-1,3	0,030
Sintomas graves de asma	6,7	6,7	0	1,000
Sibilos aos exercícios	21,4	18,7	-1,2	0,010
Tosse seca noturna	35,6	43,7	+1,9	< 0,001
Rinoconjuntivite	20,4	35.4	+5,2	< 0,001

* Teste Qui-quadrado.

Grande parte da variabilidade da prevalência do relato de asma e do diagnóstico médico deveu-se ao expressivo incremento na variação anual média no período no sexo feminino. Além disso, a prevalência de tosse seca noturna e de relato de rinoconjuntivite foi significativamente maior no sexo feminino nos dois períodos do estudo (Tabela 2).

**Tabela 2:** Prevalência e variação anual média de asma alguma vez, diagnóstico médico e sintomas no último ano e relato de rinoconjuntivite em adolescentes de 12 a 14 anos, segundo sexo. Florianópolis, SC, 2001 a 2012.

Variável (%)	2001	2012	Variação anual (%)	p*
Asma alguma vez				
Masculino	11,9	14,1	+1,6	0,090
Feminino	10,0	15,6	+4,1	< 0,001
Diagnóstico médico de asma				
Masculino	8,5	10,6	+2,0	0,070
Feminino	6,2	11,7	+5,9	< 0,001
Sibilos atuais				
Masculino	17,8	14,8	-1,7	0,030
Feminino	19,6	17,5	-1,0	0,200
Sibilos aos exercícios				
Masculino	18,8	18,2	-0,3	0,600
Feminino	23,7	19,1	-1,9	0,003
Tosse seca noturna				
Masculino	30,1	36,2	+1,7	0,001
Feminino	40,6	51,0	+2,1	< 0,001
Rinoconjuntivite				
Masculino	14,9	26,6	+5,4	< 0,001
Feminino	20,4	35,4	+5,1	< 0,001

* Teste Qui-quadrado.

A prevalência de sibilos atuais, sintomas de asma grave, sibilos aos exercícios, tosse seca noturna e relato de rinoconjuntivite foi significativamente maior nos adolescentes que referiram asma alguma vez. A maioria dos participantes que referiram asma alguma vez teve diagnóstico confirmado por médico, tanto em 2001 quanto em 2012 (Tabela 3).

**Tabela 3:** Prevalência de sintomas de asma, relato de rinoconjuntivite e diagnóstico médico de asma em adolescentes de 12 a 14 anos, que referiram ter asma. Florianópolis, SC, 2001 a 2012.

Asma alguma vez	2001			2012		

	Sim (%)	Não (%)	p*	Sim (%)	Não (%)	p*
n	333	2.720		380	2.183	
Sibilos nos últimos 12 meses	50,8	14,9	< 0,001	45,8	11,0	< 0,001
Sintomas graves de asma	25,4	4,4	< 0,001	24,2	3,7	< 0,001
Sibilos aos exercícios	48,0	18,1	< 0,001	44,2	14,2	< 0,001
Tosse seca noturna	52,6	33,5	< 0,001	59,1	41,0	< 0,001
Rinoconjuntivite	32,7	15,9	< 0,001	45,8	28,5	< 0,001
Diagnóstico médico de asma	60,7	0,8	< 0,001	68,4	1,1	< 0,001

* Teste Qui-quadrado.

Embora tenham ocorrido diferenças significativas entre a rede pública e privada nos dois períodos, a variação anual média nas diversas prevalências, com exceção de rinoconjuntivite, foi similar entre as duas redes (Tabela 4).

**Tabela 4:** Prevalência e variação anual média no período de relato de asma alguma vez, diagnóstico médico e sintomas de asma no último ano e relato de rinoconjuntivite de acordo com a rede de ensino (pública e privada). Florianópolis, SC, 2001 a 2012.

Variável (%)	2001	2012	Variação anual (%)	p*
Asma alguma vez				
Pública	9,7	13,5	+3,1	< 0,001
Privada	13,7	17,6	+2,3	0,030
Diagnóstico médico de asma				
Pública	6,1	9,9	+4,5	< 0,001
Privada	10,1	13,5	+2,8	0,030
Sibilos atuais				
Pública	18,5	14,0	-2,5	< 0,001
Privada	19,5	20,5	+0,5	0,600
Sibilos aos exercícios				
Pública	21,8	18,5	-1,7	0,009
Privada	20,4	19,2	-0,5	0,500
Tosse seca noturna				
Pública	35,2	40,9	+1,4	< 0,001
Privada	36,4	49,5	+2,8	< 0,001
Rinoconjuntivite				
Pública	16,5	27,2	+4,6	< 0,001
Privada	20,6	38,9	+6,9	< 0,001

* Teste Qui-quadrado.

## DISCUSSÃO

Os resultados mostraram aumento significativo na variação anual média da prevalência de asma e de rinoconjuntivite em adolescentes de 12 a 14 anos, no período. Esse resultado deveu-se predominantemente ao aumento de participantes do sexo feminino. Houve aumento no diagnóstico médico de asma em meninas e em ambas as redes de ensino (pública e privada). Em contraste, a prevalência de sintomas graves de asma permaneceu inalterada. A prevalência de sibilos atuais e aos exercícios, tosse seca noturna, sintomas graves de asma, rinoconjuntivite e diagnóstico médico de asma foi significativamente maior em adolescentes que relataram asma alguma vez do que naqueles que a negaram. Estes últimos resultados confirmam a consistência e confiabilidade das respostas referentes à asma e seus sintomas nos dois períodos do estudo.

Solé et al^20^ compararam a prevalência de asma e rinoconjuntivite nas Fases I e III do estudo ISAAC e analisaram as mudanças dessas condições em adolescentes entre 13 e 14 anos em cinco cidades brasileiras (Recife, Salvador, São Paulo, Curitiba e Porto Alegre). Esses autores constataram tendência à redução na prevalência de sibilos atuais, mas não na prevalência de asma e de rinoconjuntivite. A redução na prevalência de relato de asma alguma vez e sibilos dificultando a fala (um dos sintomas graves de asma) foi mais consistente em adolescentes de Porto Alegre. Houve diminuição de sibilos atuais em adolescentes de todos os locais estudados, com exceção de Curitiba e Recife.

O estudo ISAAC, Fases I e III,^4^ mostrou grandes variações de prevalência, não apenas entre diferentes países, mas também dentro de um mesmo país, em que diversos centros realizaram o estudo. Além disso, estudo mais detalhado da prevalência global dos sintomas de asma nas Fases I e III do estudo ISAAC apontou que a variabilidade na prevalência encontrada entre os diversos países deveu-se ao maior aumento anual da prevalência de asma e seus sintomas nos países onde esta prevalência era menor na Fase I; e a uma estabilização ou mesmo diminuição nos países onde esta prevalência era maior.^16^

Uma possível explicação para as diferenças de prevalência de asma e seus sintomas em Florianópolis e nas demais capitais brasileiras estudadas por Solé et al,^20^ a exemplo do estudo de Pearce et al,^16^ é que essas prevalências já eram superiores às relatadas em Florianópolis, naquelas capitais. Outras possibilidades são o intervalo entre as Fases I e III do estudo ISAAC e uma potencial interferência dos fatores locais no considerável aumento na prevalência de asma e de rinoconjuntivite em nosso estudo. Esses fatores podem relacionar-se com mudanças socioeconômicas, maior atenção aos sintomas de ambas as doenças por parte de pacientes, familiares e médicos, ou ainda, por outros fatores não detectados neste estudo, o que merece ser investigado no futuro. O interstício de 10 anos entre os dois estudos pôde ser caracterizado pelo aumento da oferta e do acesso aos serviços de saúde, além de aumento substancial da densidade demográfica, com suas repercussões, notadamente na esfera ambiental.

Wehrmeister et al,^24^ usando dados de prevalência da pesquisa nacional por amostra de domicílios, estudaram a tendência da prevalência de asma no Brasil em crianças e adolescentes de 10 a 19 anos, de 1998 a 2008. O aumento na prevalência de asma foi 2,2% ao ano no grupo de adolescentes. Os maiores incrementos foram observados entre os meninos e entre moradores da zona rural. Os resultados do presente estudo diferem daqueles acima citados, uma vez que o maior aumento anual no período na prevalência de asma ocorreu em meninas. Diferenças metodológicas entre os dois estudos podem explicar essas discrepâncias.

Outro estudo brasileiro, usando a metodologia ISAAC em adolescentes com 13 e 14 anos, relatou maior prevalência de sintomas de asma no sexo feminino.^12^ Vários fatores são relacionados à associação de asma na adolescência e sexo feminino, incluindo fatores genéticos, interações materno-fetais, maturação do sistema imune, alterações hormonais e influências psicossociais e ambientais próprias de cada sexo.^1^ ^,^ ^12^

Apesar do expressivo aumento do relato de asma alguma vez e de diagnóstico médico de asma no presente estudo, houve diminuição na prevalência de sibilos atuais (nos últimos 12 meses) e sibilos aos exercícios na análise global dos resultados e na comparação da distribuição deste sintoma por sexo e por rede de ensino. A diminuição significativa desses sintomas, sem acompanhar a diminuição do diagnóstico de asma, pode relacionar-se à maior disponibilização de medicamentos para asma na rede pública, bem como, maior acesso aos serviços de saúde na última década. Da mesma forma, a estabilização da prevalência de sintomas graves de asma pode decorrer dos mesmos fatores.

A tendência mundial de sintomas e de presença de rinoconjuntivite utilizando os dados dos estudos ISAAC Fase I e III foi estudada por Asher et al.^4^ No Brasil, esses autores constataram que a prevalência média foi de 16,2% na Fase I e de 15,8% na Fase III do estudo. Mais especificamente, ocorreram variações importantes nas diversas cidades avaliadas e a prevalência de rinoconjuntivite foi de 25,0% e 21,1% em Salvador, de 11,3% e 14,2% em Recife, 12,6% e 15,6% em São Paulo, 14,1% e 17,0% em Curitiba e de 17,6% e 14,2% em Porto Alegre, respectivamente nas Fases I e III.^8^ Os resultados do presente estudo mostraram incremento na variação anual superior aos relatados na literatura.^6^ ^,^ ^7^ Essas diferenças podem ser devidas ao intervalo de anos estudados ou a mudanças socioeconômicas.

A asma e rinoconjuntivite são condições comumente associadas. Os principais fatores etiológicos para ambas são a atopia, exposição a alérgenos dentro e fora de casa ou no trabalho, além de fatores como: variações geográficas, genética, estilo de vida, industrialização, pobreza, convívio com animais, tabagismo passivo, umidade doméstica, e infecção viral durante os primeiros anos de vida.^13^ No presente estudo, 32,7% e 45,8% dos adolescentes que referiram ter asma alguma vez em 2001 e 2012, respectivamente, relataram ter rinoconjuntivite.

Diversos aspectos do presente estudo conferem robustez aos resultados: uso de uma metodologia padronizada nos dois períodos da pesquisa; elevado índice de resposta; rigorosa checagem dos dados obtidos e da metodologia de sua aquisição.

Uma possível limitação deste estudo foi a inclusão de indivíduos de 12 anos, uma vez que a quase totalidade dos estudos ISAAC relatam resultados para adolescentes com idade entre 13 e 14 anos. A decisão em manter os alunos com 12 anos por completarem 13 anos no ano do estudo foi igualmente mantida nos dois períodos.

No entanto, isso parece ser menos importante do que a representatividade da amostra, já que os próprios entrevistados não são os mesmos. Em outros relatos do estudo ISAAC Fases I e III em alguns centros, as escolas entrevistadas para a Fase III foram completamente diferentes das escolas da Fase I.^4^

Os resultados do presente estudo mostram que houve aumento anual significativo no período na prevalência do relato de asma e de rinoconjuntivite e seus sintomas em adolescentes entre 12 e 14 anos de Florianópolis de 2001 a 2012. Houve aumento do diagnóstico médico de asma, porém não acompanhado de aumento da sintomatologia considerada grave, o que pode explicar a melhora na qualidade do atendimento médico no diagnóstico e nos aspectos terapêuticos. Porém, essa evolução está acima da observada em outros países e no Brasil, sugerindo que fatores locais podem estar envolvidos e merecem ser investigados em estudos futuros.

## Footnotes

a Piazza HE. Prevalência de sintomas de asma e rinite em adolescentes escolares da cidade de Florianópolis [dissertação]. Florianópolis (SC): Universidade Federal de Santa Catarina; 2001.
